# Ecological traits, genetic diversity and regional distribution of the macroalga *Treptacantha elegans* along the Catalan coast (NW Mediterranean Sea)

**DOI:** 10.1038/s41598-020-76066-6

**Published:** 2020-11-05

**Authors:** Alba Medrano, Bernat Hereu, Simone Mariani, João Neiva, Marta Pagès-Escolà, Cristina Paulino, Graciel·la Rovira, Ester A. Serrão, Cristina Linares

**Affiliations:** 1 Department of Evolutionary Biology, Ecology and Environmental Sciences, Institut de Recerca de La Biodiversitat (IRBIO), University of Barcelona, Av. Diagonal 643, 08028 Barcelona, Spain; 2 Centre d’Estudis Avançats de Blanes – CSIC, Accés Cala Sant Francesc 14, Blanes, 17300 Girona, Spain; 3 Center of Marine Science (CCMAR), University of Algarve, Campus de Gambelas, 8005-139 Faro, Portugal

**Keywords:** Ecology, Marine biology

## Abstract

The widespread decline of canopy-forming macroalgal assemblages has been documented in many regions during the last decades. This pattern is often followed by the replacement of structurally complex algal canopies by more simplified habitats (e.g., turfs or sea urchin barren grounds). Against all odds, the fucoid *Treptacantha elegans*, a large Mediterranean brown macroalga, broadened its depth range to deeper and exposed environments and displayed an unexpected range expansion along the northern coast of Catalonia over the last two decades. Here, we reconstruct the spread of *T. elegans* in time and space and unravel ecological and demographic traits such as population dynamics and genetic patterns to provide a comprehensive and integrated view of the current status and geographical expansion for this species. Fast-growing dynamics, early fertile maturity, and high turnover rate are the main competitive advantages that allow the exposed populations of *T. elegans* to colonize available substrata and maintain dense and patchy populations. We also provided evidence that the deeper and exposed populations of *T. elegans* constitute a single group across the Catalan coast, with little genetic differentiation among populations. This seems to support the hypothesis of a unique source of spread in the last decades from the Medes Islands No-Take Zone towards both southern and northern waters.

## Introduction

How marine populations persist, evolve, and change their geographical distributions as a response to global change is one of the main questions in contemporary ecology^1,2^. Species may redistribute to keep their preferred environmental conditions^3–6^ or adapt their physiology as a response to global and local stressors^5,7^. Macroalgae are dominant primary producers and ecosystem engineers that provide structure and ecosystem services (e.g., food and fisheries production and carbon storage)^8–10^ to many temperate and sub-temperate shallow coastal habitats where they play a key role in ecosystem functioning^11,12^. As a response to global and local stressors (i.e., ocean warming or coastal pollution), they may show both geographical expansions, when species colonize new habitats, or contractions, when populations disappear from areas previously inside their distributional range^4,13^.

In the Mediterranean Sea, *Cystoseira *sensu lato fucoids (here used to designate Atlantic-Mediterranean fucoid species of *Cystoseira *sensu stricto, *Carpodesmia* and *Treptacantha*^14^) are late successional species and among the major canopy-forming macroalgae. They provide three-dimensional structure, food, and shelter to many associated species^9,15^. Decline of these species may drive to a decrease in habitat complexity with important consequences for benthic biodiversity and ecosystem functioning^10,15–18^. All *Cystoseira *sensu latospecies, except *C. compressa*, appear in the Annex II of the Barcelona convention (2010) among those taxa that are considered threatened or endangered and need protection measures^19^. *Cystoseira *sensu lato species show limited dispersal abilities and propagules often disperse over a few meters^19,20^. This leads to the existence of monospecific stands near parent populations^21^. This trait combined with local environmental factors may explain the lack of connectivity for many populations of *T. elegans* in different areas of the Mediterranean Sea^22,23^, something that seriously jeopardizes their conservation. Nonetheless, the few studies on population genetics suggest that water currents may play a significant role in long-distance dispersal (presumably via drifting of detached reproductive branches) of *Cystoseira *sensu lato populations^24–26^. Thus, despite the well-documented general decline of local populations^16,18,27^, evidences for stability and even expansion have been also reported in some areas^27–29^.

*Treptacantha elegans* (Sauvageau) Orellana and Sansón (until very recently *Cystoseira elegans*^14^) is a Mediterranean endemic species. The Mediterranean Sea is oligotrophic, with relatively high salinity, high summer temperatures, and extremely reduced tides, which are mainly regulated by the atmospheric pressure^30^. *T. elegans* was first described by Sauvageau for Banyuls-sur-Mer (South-Eastern France)^31^. This species typically inhabits shallow waters down to 2–3 m depth^31^, where forms extensive stands at the innermost rocky bottoms of coves and other shallow environments characterized by low to medium water movements^32^. Recent studies have reported a significant regression of *T. elegans* in shallow and sheltered environments from the N–W Mediterranean Sea^16,18,23^. However, in the past two decades, some populations of *T. elegans* (identification by M. Verlaque, personal communication) have expanded their distribution in the Medes Islands No-Take Zone (NTZ) and the nearby areas (North-Western Mediterranean Sea), where the species can be now found in wave-exposed habitats located between 5 and 15 m depth, with some sparse thalli at 20 m depth. The reasons for this pattern are yet to be determined.

Here we investigate this recent spread throughout the North Catalan coast. Concretely, our goals are: (1) provide insights about the main traits of deeper and exposed populations of *T. elegans* and the species population ecology; (2) assess its historical and current distribution in the Catalan coast; and (3) provide an estimate of the patterns of genetic diversity and differentiation among populations including ancient and more recently established zones.

## Results

### Phenological and ecological traits

The seawater temperature in the north Catalan coast is characterized by strong seasonal variability. In 2018, the mean annual temperature at 5 m depth was 17.27 °C ± 1.26 (Mean ± SE). In accordance with the well-known thermal regime of this area^33^, minimum winter temperatures were recorded in February and March (12.35 °C ± 0.3, Mean ± SE) and the maximum in August (23.82 °C ± 0.9, Mean ± SE) just before the gradual drop throughout autumn.

**Figure 1:**
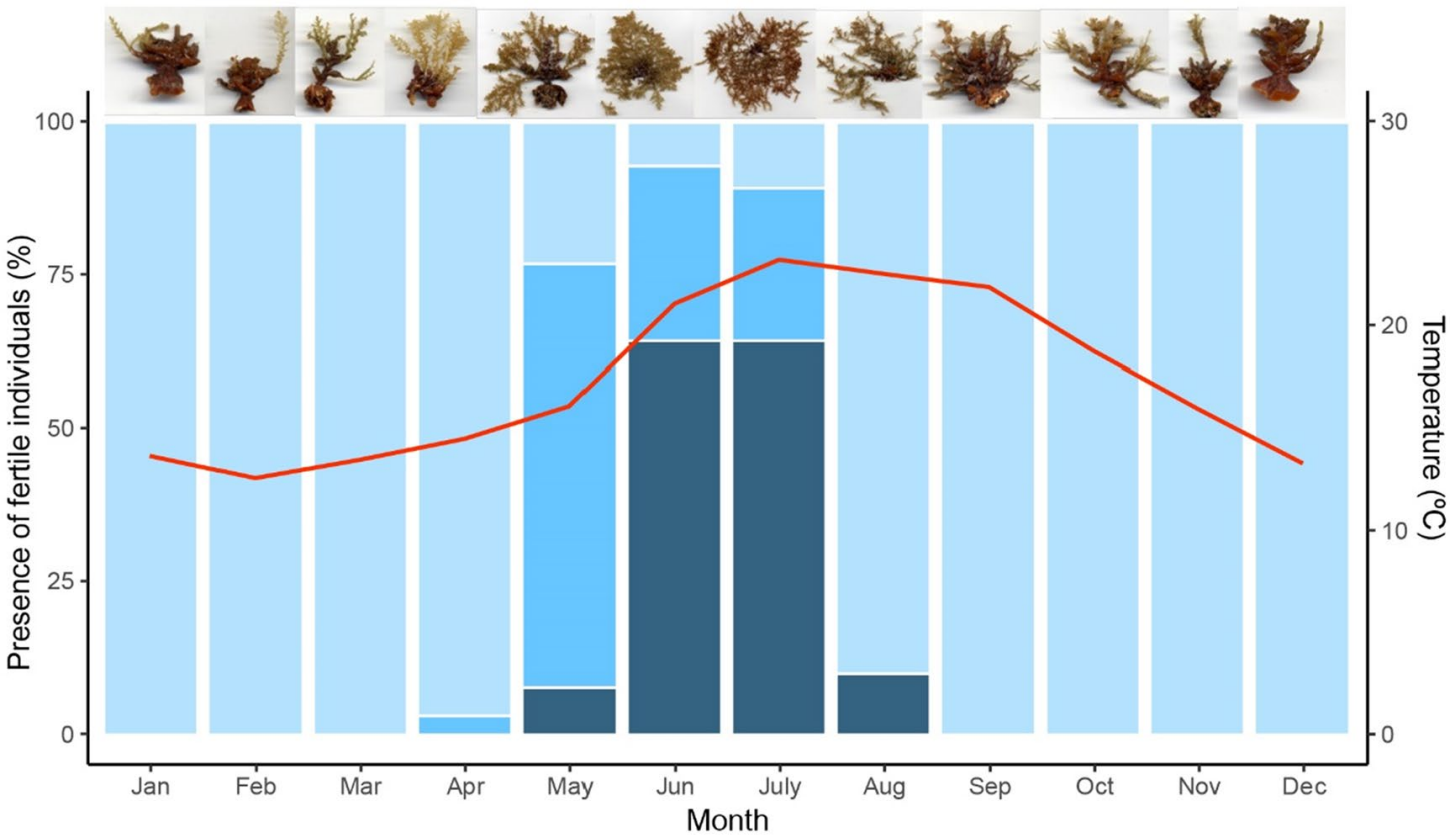
Monthly presence of fertile *Treptacantha elegans* individuals. Light blue bars represent the percentage of immature individuals, without the presence of reproductive structures. Medium-blue bars represent the percentage of the individuals beginning the reproductive stage, with reproductive receptacles in the maturing process. Dark blue bars represent the percentage of fertile and reproductive individuals. The red line shows the mean monthly temperature at 5 m depth. Pictures at the top illustrate the monthly canopy-forming branches development stage.

Branches of *Treptacantha elegans* from deeper and exposed infralittoral habitats (5–15 m depth) began to grow from the perennial basal disc (or holdfast) in early spring when sea water temperature starts to rise (Fig. 1). The receptacles located at the base of each spiny branch began to mature progressively as branches approached their maximum development. The reproductive period spanned from the end of May to August, individuals being fertile mainly when water temperature rose above 18 °C. In June and July, 90% of the sampled individuals showed receptacles (Fig. 1). All fertile individuals measured more than 4.5 cm. Like the thalli from more sheltered environments, specimens of *T. elegans* from deeper, exposed habitats shed their branches after the reproductive period and remained attached to the substrate through the perennial holdfast until the following spring^34,35^.

**Figure 2:**
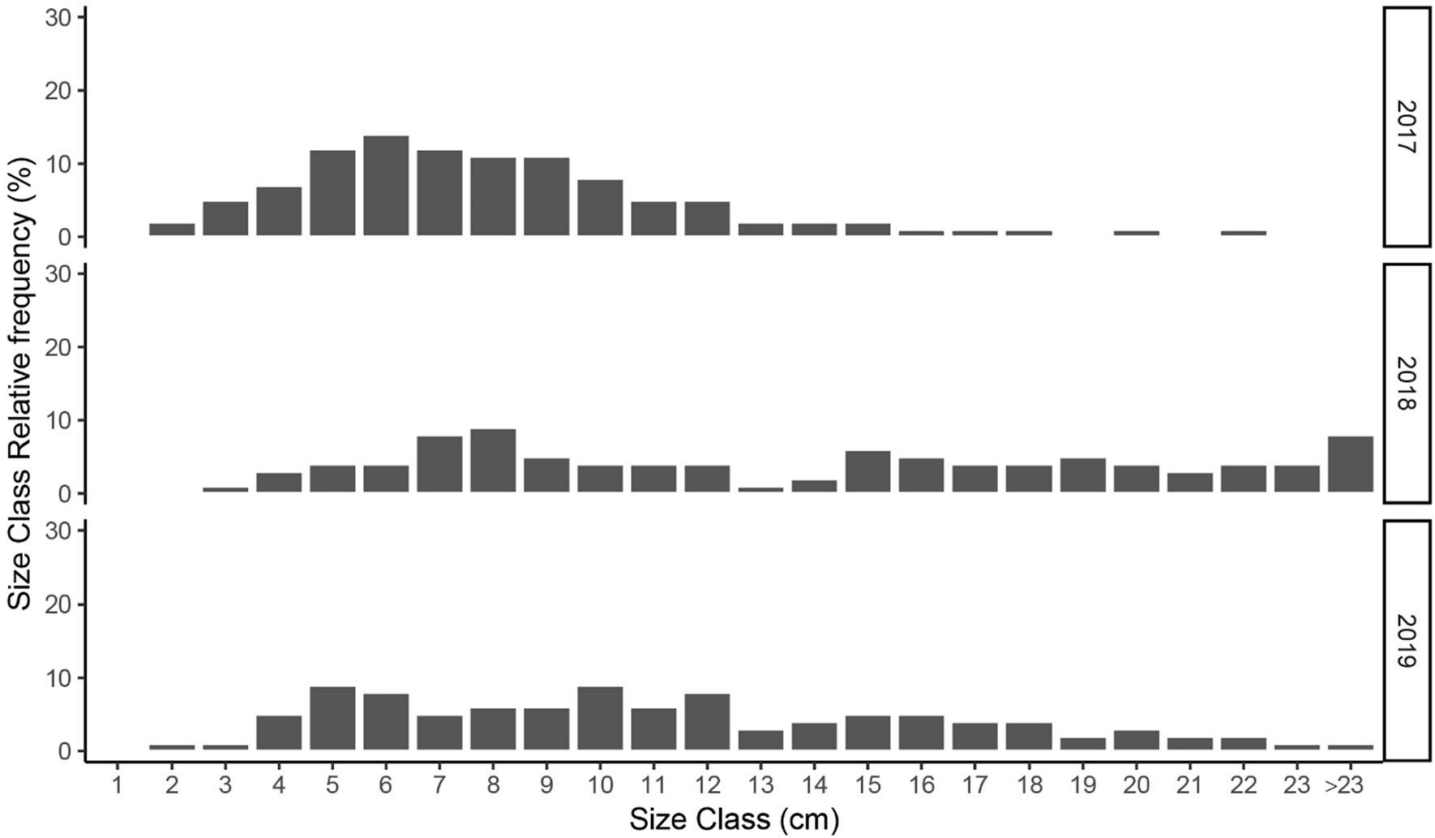
Size-class distribution of *Treptacantha elegans* populations across the three studied years. Relative frequency of size-classes was estimated on 1 cm intervals (length of the longest axis).

**Figure 3:**
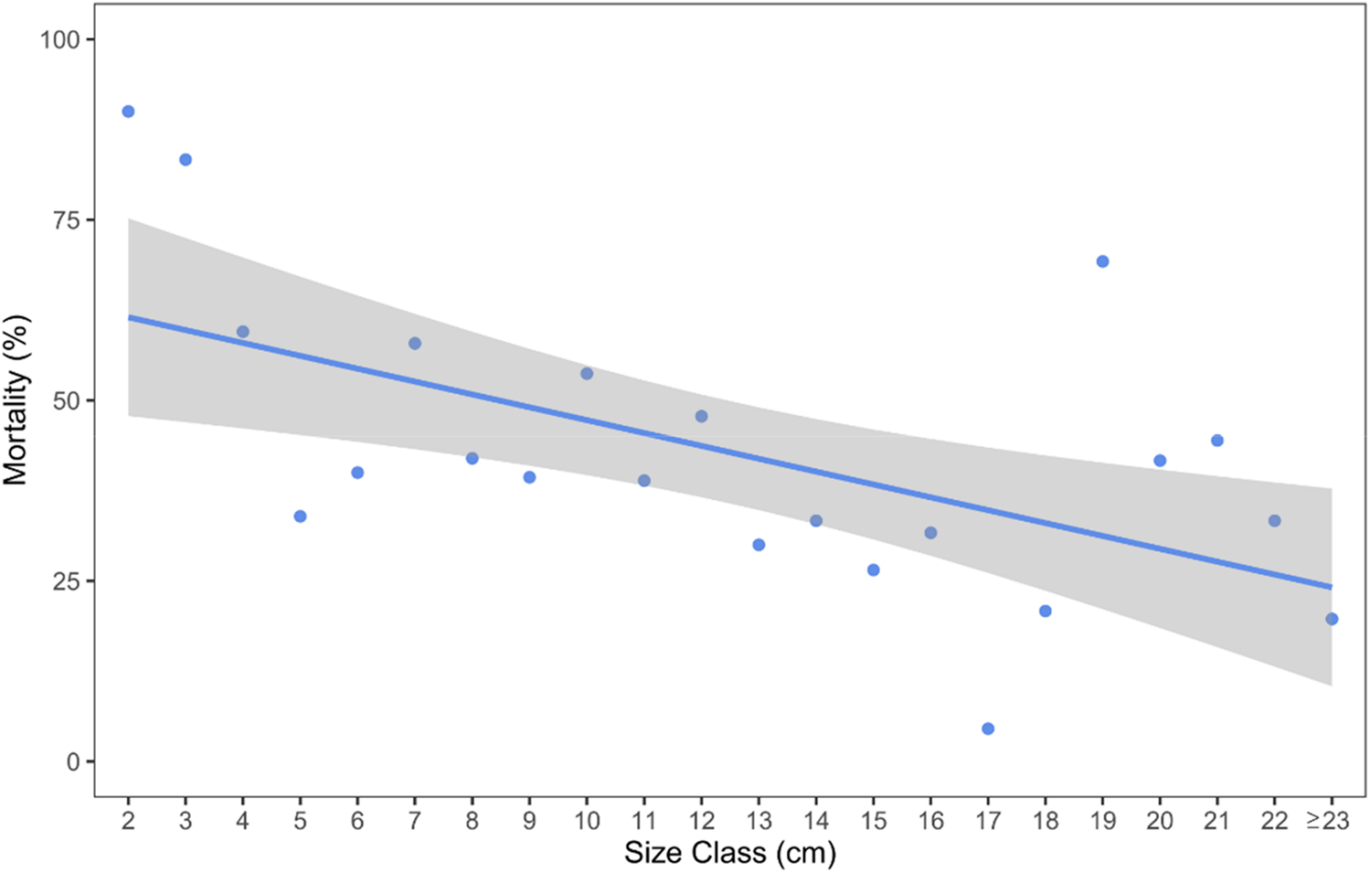
The relation between the mortality rate and the size of the *T. elegans* stands. The blue line represented the generalized linear model fitted between the response variable and the predictor fitted to the most likely distribution (negative binomial).

The deeper, exposed populations monitored in the Medes Islands showed similar densities (51.3 ± 4.26 ind/m^2^, Mean ± SE) over the three years. No differences were found in density (χ^2^_2_ = 4.8, *p* = 0.09) and mean size of individuals (χ^2^_2_ = 3.7, *p* = 0.16) across the three sites. When grouped into 1 cm size classes, the size distribution of *T. elegans* individuals mostly ranged from 1 to 23 cm (Fig. 2) but unusual larger sizes (up to 42 cm), like those found in specimens from shallow, sheltered environments^36^, were also found. The mean size of *T. elegans* individuals sampled over the 3 years in the 6 permanent plots was 10.92 ± 0.21 cm (Mean ± SE). The mean growth rate within first-year individuals was 8.6 cm ± 0.6 (Mean ± SE). Some specimens could reach relatively large sizes, being 28 cm the maximum growth observed for an individual recruited the previous spring. New, annually appeared recruits were about one-third of the total counts in the permanent plots (33.7% ± 7.9, Mean ± SE), 82% of which exceeded the fertile minimum size (4.5 cm). Mean annual mortality rate within the 3 populations was about 36% (35.9% ± 9.8, Mean ± SE). Small specimens showed higher annual mortality rates than the larger ones (Fig. 3, Table 1).

**Table 1:** Results of the generalized linear model (GLM) testing differences in the *T. elegans* mortality rates between the size classes.

Model	Variables	Coefficients				AIC
		Estimate	SE	*z *value	*p*	
Mortality rate ~ size class	Intercept	0.6618	0.0759	8.714	< 0.001	− 2.492
	Size class (cm)	− 0.0192	0.0056	− 3.430	** < 0.001**	

Significant differences (< 0.001) were indicated in bold.

### Spatial and temporal distribution

**Figure 4:**
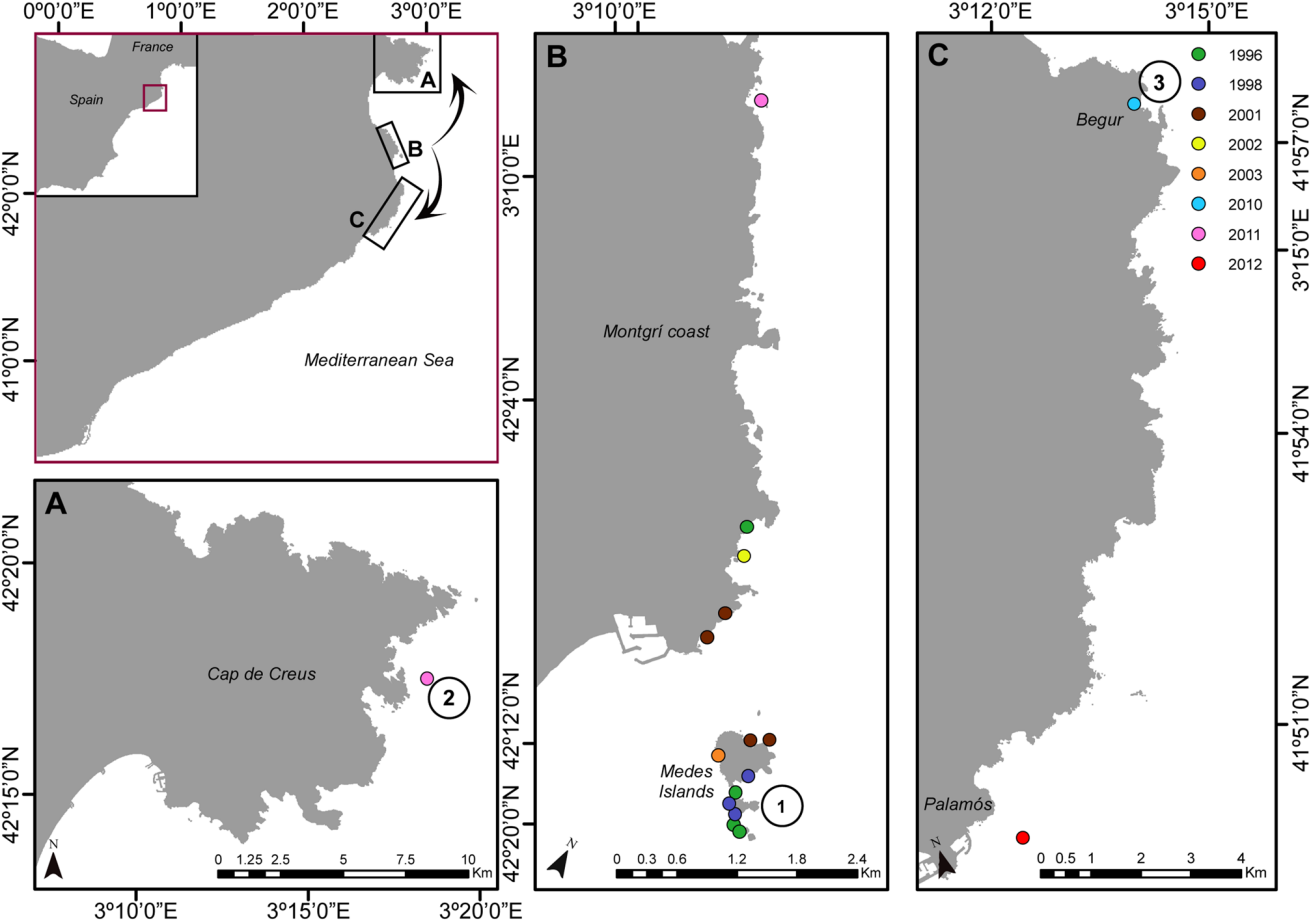
Geographical distribution of deep *Treptacantha elegans* over the studied years in the Catalan coast. Colored dots indicate the temporal sequence of their first report and circled numbers identify the populations where molecular analyses were conducted. Map was created using ArcMap 10.7 (https://desktop.arcgis.com/en/arcmap).

Since their first observation in the Medes Islands NTZ^37^, *T. elegans* populations from deeper and exposed shores have progressively expanded to other areas of the coast of Catalonia (Fig. 4). The farthest populations were detected 30 km away from the Medes Islands in 2011 and 2012 (Table 2). All the 17 populations detected over the last 2 decades were still present in 2018 on rocky and exposed bottoms between 5 and 15 m depth.

**Table 2:** Details of historical information on *T. elegans* compiled for the present study. (*) CEAB (Centre d'Estudis Avançats de Blanes; CSIC), data have been collected since 1992 by the Macroalgae and invasive species monitoring group.

Locality	Distance from Medes Islands (km)	GPS coordinates	Year of first observation	References	Type of document	Previous studies in the area?	Current distribution
Ferranelles	0	42.04195; 3.22577	1996	^37^	Article	Yes	Present
Tascons	0	42.0416; 3.2268	1996	^37^	Article	Yes	Present
Freueto	0	42.0446; 3.2241	1996	^37^	Article	Yes	Present
Falaguer	2	42.0659; 3.2096	1996	^37^	Article	Yes	Present
Cova de la Reina	0	42.0464; 3.2244	1998	^72^	Article	Yes	Present
Sant Estiu	0	42.0428; 3.2253	1998	^72^	Article	Yes	Present
Raco Portitxol	0	42.0434; 3.2241	1998	^72^	Article	Yes	Present
Salpatxot	0	42.0492; 3.2226	2001	^73^	Article	Yes	Present
P.Deu	0	42.0507; 3.2245	2001	^73^	Article	Yes	Present
Molinet	1	42.0555; 3.2120	2001	^73^	Article	Yes	Present
Arquets	1.2	42.0582; 3.2125	2001	^73^	Article	Yes	Present
Dui	1.7	42.0635; 3.2113	2002	^73^	Article	Yes	Present
Embarcador	0	42.0466; 3.2201	2003	CEAB*	Report	Yes	Present
Aiguafreda, Begur	9	41.9641; 3.2277	2010	CEAB*	Report	Yes	Present
Messina, Cap de Creus	29	42.2912; 3.3083	2011	CEAB*	Report	Yes	Present
Baix de Cols	7.5	42.1001; 3.1861	2011	CEAB*	Report	Yes	Present
Llosa, Palamós	25	41.8461; 3.1482	2012	CEAB*	Report	Yes	Present

### Population genetic structure and diversity

**Figure 5:**
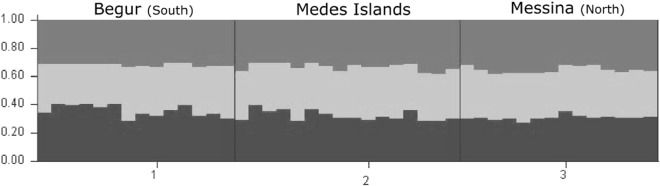
Genetic subdivision of *Treptacantha elegans* populations based on STRUCTURE, assuming 3 genetic clusters. Each vertical line represents the proportion of genome assign to each cluster for each individual. Black lines separate different populations. Note the absence of any population differentiation.

The 8 microsatellite loci showed moderate polymorphism, ranging from 3 to 14 total alleles per locus (64 in total). Allelic richness was higher in the Medes Islands NTZ population and lowest in the northern population (Messina; Table 3). Although it showed the lowest number of private alleles, marginally higher genetic diversity was also observed in Medes Islands NTZ (Table 3). All populations showed positive inbreeding coefficients (F_IS_) resulting from heterozygote deficiency (Table 3). Mean F_ST_ across all populations and loci was 0.07. The analyses of Molecular Variance (AMOVA) revealed that most genetic variation occurred within populations (93%, Supplementary Table S1). Pairwise F_ST_ estimates revealed no differentiation between the Medes Islands and Begur (separated only by 9 km, Fig. 4 locations 1 and 3). Pairwise estimates between the Medes Islands with Messina and Messina with Begur were significant (Table 4). According to Structure Harvester the best K was 3. Structure results showed negligible population differentiation within *T. elegans* populations across the Catalan coast, i.e., revealed a genetically homogeneous region (Fig. 5, Supplementary Table S2).

**Table 3:** Multilocus genetic diversity estimates for *T. elegans* populations (n = 20) across Catalan coast based on 8 microsatellite loci. For A, H_e_, and H_o_ values are mean ± SE over all 8 loci. H_e_ corresponds to unbiased expected heterozygosity^74^. cF_IS_ corresponds to the inbreeding coefficient after correction for null alleles.

Location	Map code	Allelic richness (A)	Expected heterozygisity (H_e_)	Observed heterozygosity (H_o_)	Inbreading coefficient (cF_IS_)	Number of private alleles
Messina	2	4.5 ± 0.5	0.7 ± 0.03	0.39 ± 0.09	0.34	6
Medes Islands	1	6 ± 1.2	0.74 ± 0.05	0.56 ± 0.1	0.11	4
Begur	3	5.625 ± 0.8	0.72 ± 0.1	0.49 ± 0.16	0.31	8

**Table 4:** Pairwise F_ST_ estimates between the three populations over 8 loci.

Populations	F_ST_		Distances (km)	
	Medes Islands	Begur	Medes Islands	Begur
Messina	0.073*	0.099*	29	37
Medes Islands		0.030		9

Asterisks indicate significant values of F-statistics (*p* < 0.05) for 1023 permutations.

## Discussion

Most species of *Cystoseira *sensu lato are often considered late successional species, are especially sensitive to environmental and man-induced stressors^38^, and show limited dispersal abilities^19,21^. For these reasons, they can be considered less competitive species which makes the results of this study surprising. Despite its decline in shallow and sheltered environments from neighboring areas^16,18^, the brown alga *Treptacantha elegans* has remarkably increased both its depth range and spatial distribution over the last two decades along the Catalan coast.

A better knowledge of the population dynamics of a species is crucial for understanding, explaining, and predicting species distributions. In the Medes Islands NTZ, deeper and exposed *T. elegans* forests showed fast-growing dynamics. During their first year of life, more than 80% of their stands were capable to reach fertile maturity and the relatively largest sizes that may ensure survival rates higher than 50%. These uncommon traits among species of the same genus clearly represent an advantage for colonizing new available substrates. The observed similar mean annual mortality and recruitment rates suggest a high population turnover rate, which might also represent a major advantage to *T. elegans.*

Our molecular analyses of the three exposed and deeper *T. elegans* populations across the Catalan coast showed great genetic homogeneity despite relatively high intra-population variation and inbreeding. The first *T. elegans* population from exposed shores was detected in the Medes Islands NTZ in 1996 and its origin has been thought to be related to the protection effect^39^, specifically to the lower density of herbivores in the reserve^40^. Since then, only a slight expansion (< 1 km) has been observed during the following 15 years. The southern (Begur) and northernmost (Messina) populations analyzed here have not been described until 2010 and 2011 respectively, even though regular monitoring was carried out in these locations. Hence, we assume that the Medes Islands may represent the source population from which the species expansion across the exposed shores in the Catalan coast begun. The Medes Islands population also exhibited the highest allelic richness, which supports our assumption of it being a mature population thus a reasonable source population. As in other *Cystoseira *sensu lato species, our results may suggest a common ancestral gene pool but loosely connected populations^22,26,41^. This is supported by the patchy distribution of *T. elegans*, a pattern likely due to the scattered presence of rocky and suitable habitats in the area and the limited dispersal range of gametes in fucoids^19,20,21,42^. Unfortunately, our data are not sufficient to detect a clear correlation between distance and genetic differentiation within populations.

While the dispersal distance of early life stages is less than 10 m from source populations in *T. elegans*^43^, here we show the existence of two populations 30 km away from the Medes Islands NTZ. This may be explained by the fact that this species, as other fucoids, is capable to disperse through detached or drifting fertile parts of mature individuals^21,27,44^ or by animals^18^. This may explain the southern spreading through the Northern Current (NC), which flows with a permanent southwestward circulation in the Northwestern Mediterranean basin^45^. Although a NC mediated spread in the opposite direction is unlikely, other processes such as wind-induced currents or extreme meteorological conditions play important roles in the water circulation of this complex hydrodynamical region. This may explain the passive transportation of *T. elegans* from the exposed shores of the Medes Islands to the northern studied exposed areas^46^.

Increased tolerance and adaptation and/or migration to different locations within their specific niche are the main responses to environmental changes in macroalgae^7^. Morphological differences have been reported in *T. elegans* individuals between populations from sheltered and exposed infralittoral habitats^23^ such as the presence of thicker and spinier branches in the individuals from exposed shores, a trait that may be the result of a phenotypic plasticity response (acclimation rather than adaptation) to such environments, as no evidence for genetic differences has been detected in preliminary assays of these morphotypes (Authors, unpublished data). The potential source of the first deeper *T. elegans* population observed in the exposed shores of the Medes Islands is unknown. However, it may have arrived from the original shallow population in Banyuls-sur-Mer (France), where this species was described for the first time^31^, or from other shallow littoral populations from the north of Catalonia where the species was more abundant in the past^23^.

Several environmental drivers and the interaction of some favorable conditions may have promoted the spread observed. Seawater eutrophication is one of the major causes for the disappearance of *Cystoseira *sensu lato populations in the Mediterranean Sea^29,47^. Evidence of a general improvement in coastal water quality, particularly during the 1990s^48^, is a possible explanation for the expansion of *Cystoseira *sensu lato species in the studied area^49^. Besides, the Medes Islands NTZ might have worked as a refuge for *T. elegans* populations during the period of improvement of environmental conditions, mainly because of the lower density of herbivores due to the predatory fish control in the reserve^40^. In addition to these local stressors, global warming and extreme climatic events (such as storms and heatwaves) are also threatening marine coastal life, especially in shallower waters^50^. The increase in sea-surface temperature over the last decades^51,52^ may have led to species responses in macroalgal assemblages such as the observed here^53^. While we know that *T. elegans* may withstand high summer temperatures as an adaptation to the shallow environment^23^, our results suggest that a shift in the depth-range distribution and a subsequent acclimation to the new environments may be a result of warming.

Our results shed new light on the ability of a *Cystoseira *sensu lato species to persist or even widen their geographical distributions under global change scenarios. For this to happen, however, it is important to improve the water quality and to protect population sources that ensure dispersal like those found in the Medes Islands NTZ, especially in organisms with short-dispersing propagules. Finally, given its fast and stable population dynamics (early fertile maturity and high turnover rate), we highlight that *T. elegans* represent an ideal organism to conduct habitat restoration actions in previously degraded systems such as sea urchin barrens or turf-dominated habitats^43^.

## Methods

### Phenology and demographic data

Measurement of the main axis has been reported as a good indicator of algae size in morphometric studies of other *Cystoseira *sensu lato species^54^. However, as *Treptacantha elegans* is characterized by a tiny main axis (1–3 cm length^55^), this trait hinders accurate measurements in situ unless invasive techniques are used. To discern the best less invasive size indicator for in situ measurements, we collected 27 specimens of Medes Islands NTZ and measured several morphometric parameters. Correlation analyses between the parameters showed high correlation between the maximum length of the longest axis and the rest of the morphometric variables measured (Supplementary Table S3). The maximum length of the longest axis was then selected as the best parameter for easy and robust measurements from different observers.

To accurately describe the *T. elegans* annual life cycle (i.e. growth of branches, reproductive period, and branch shedding) throughout the annual temperature cycle, 20 *T. elegans* individuals covering a wide size range (2–23 cm) were sampled monthly in the Medes Islands NTZ in 2018. Their fertile maturity status was discerned by pooling the individuals sampled within three stages: (1) immature individuals without receptacles; (2) individuals beginning their reproductive period, with receptacles but still immature; (3) fertile and reproductive individuals. Annual seawater temperatures were assessed by high-resolution hourly temperature recordings at 5 m depth in the Medes Islands obtained from the T-MEDNet platform (https://www.t-mednet.org).

To untangle the population dynamics of *T. elegans* from exposed shores (5–15 m depth), we performed a survey at the Medes Islands NTZ in 2017, 2018, and 2019 on three populations. These populations thrive a few hundred meters apart (see the 3 green dots in the Medes Islands, Fig. 4B). All three sites were south facing and showed healthy populations of *T. elegans* of similar extension thriving on top of the rocky boulders. At each site, two permanent plots of 1–1.5 m^2^ (depending on boulder size) were placed a few meters apart at around 10 m depth*.* Thanks to the high density of *T. elegans* populations in the patches, these tiny 6 permanent plots allowed to monitor 684 specimens. Generalized linear mixed models (GLMMs) with Poisson error distribution were applied to test the similarity of the three selected sites. Density and mean size of *T. elegans* individuals in 2017 were treated as independent response variables, the site as the fixed response variable and the plots as the random response variable. For the two fitted models we applied a Type II Wald χ^2^ test to show a non-significant effect of sites. This led us to pool together all specimens from the three sites of the Medes Islands location (Fig. 4B), which also correspond with the oldest and most mature *T. elegans* populations found in this study. All specimens in the 6 permanent plots were mapped and individually monitored yearly from 2017 to 2019 in early summer during the macroalgal maximum development period. All specimens inside the plots were measured using a caliper by SCUBA divers. Population density and size structure were calculated by pooling the individuals into 0.9 cm binned size intervals^54,56,57^. Differences in density measures between two consecutive annual surveys within the studied period were used to estimate the mortality and recruitment rates. For each survey transition, mortality was quantified as the disappearance of individuals between census and, recruits were identified as new individuals appearing in the permanent plots at each census regardless of their size. Mortality rates for each size class were compared using a generalized linear model (GLM) fitted with a negative binomial distribution and a logarithm link function after visually checking the most likely distribution of the data and residuals^58^. GLM analyses were conducted using the package “MASS” for R software^59^ and GLMMs using the package “lme4”^60^ with R version 3.3.3^61^.

### Spatial and temporal distribution

Past and present distribution of shallow (≤ 1 m depth) and sheltered *Treptacantha elegans* in the Catalan coast has been recently reported^23^. Although descriptive studies have been carried out in the shorelines here investigated since the seventies^62,63^, *T. elegans* was not found in deeper and more exposed environments until the nineties, when Sala reported its presence^37^ (as *Cystoseira spinosa*^23^) for the Medes Islands. All available information about the spatial and temporal distribution of this species along the Catalan coast is reported in Table 2.

### Molecular analysis

Samples for molecular analysis were collected in three study locations along the Catalan coast, thus covering the whole distribution range for the study area (Fig. 4, locations: 1 Medes Islands NTZ, 2 Messina, and 3 Begur). We collected twenty *Treptacantha elegans* samples corresponding to branches from 20 individuals haphazardly selected at each location, at a maximum distance among samples of 10 m. The samples were individually preserved by drying them in silica gel until DNA extraction.

Genomic DNA for each population was extracted from the dried samples using the NucleoSpin Plant II kit (Macherey–Nagel Duren, Germany) according to the manufacturer’s protocol. Eight microsatellite loci formerly developed for *Treptacantha baccata* (unpublished data from Biogeographical Ecology and Evolution research group, CCMAR) were used for genotyping. Polymerase chain reactions (PCRs) were performed in 15 µL total volume containing 1· GoTaq Flexi buffer (Promega, Madison, WI, USA), 1.5 mm MgCl_2_, 0.07 mm each dNTP, 0.4 µM of labelled (FAM, ATT0-550, ATT0-565 or HEX) forward primers, 0.4 µM of reverse primers, 1U GoTaq Flexi DNA Polymerase (Promega), and 5 µL of 1:100 diluted DNA template. In all PCRs, an initial denaturation step (95 °C, 2 min) was followed by 30 cycles of 95 °C for 30 s, a primer specific annealing temperature (Ta) for 30 s and 72 °C for 30 s, ending with a final elongation step at 72 °C for 5 min. Amplified fragments were separated using an ABI PRISM 3130 xl (Applied Biosystems, CCMAR, Portugal) automated capillary sequencer. Alleles were manually scored in STRand^64^ using the GeneScan 500 LIZ dye size standard (Applied Biosystems).

All individuals with missing data at three or more loci were excluded from analyses. These corresponded to 4 individuals, 2 from the southern population (Begur) and 2 from the norther one (Messina). Identification of genotyping errors due to null alleles, large allele dropout and the scoring errors were screening with the MICRO-CHECKER software v. 2.2.3^65^. When evidence of null alleles was significant, a correction for null alleles was applied. Estimates of genetic diversity were calculated for each locus and population using the GENETIX software v. 4.05^66^. This included the mean number of alleles per locus (allelic richness), the non-biased expected heterozygosity (H_E_), the observed heterozygosity (H_O_), the number of private alleles, and the inbreeding coefficient (F_IS_). Pair-wise differentiation, as assessed with F_ST_^67^, was calculated. Analyses of molecular variance components (AMOVA) were conducted using ARLEQUIN v3.1^68^.

Population structure was inferred using STRUCTURE v2.444^69^ considering admixture and no a priori population assignments. The correlated allele frequency model was run with a burning time of 250,000 repetitions, 1,000,000 iterations and considering a range of genetic clusters (K) from 1 to 5. The model was run 14 times for each K to check the consistency. The best K was estimated using the program Structure Harvester^70^ which ranks K’s according to Evanno method^71^.

## Supplementary information

Supplementary information.

## Data Availability

The datasets generated for this study are available on request to the corresponding author.
