# Supplementary material for: Ecological traits, genetic diversity and regional distribution of the macroalga Treptacantha elegans along the Catalan coast (NW Mediterranean Sea)
Source: Sci Rep. 2020 Nov 5;10:19219. doi: 10.1038/s41598-020-76066-6 (PMC7644675; doi:10.1038/s41598-020-76066-6)
Supplement: Supplementary file 1 — Supplementary information. [file 41598_2020_76066_MOESM1_ESM.docx]

**Ecological traits, genetic diversity and regional distribution of the macroalga *Treptacantha elegans* along the Catalan coast (NW Mediterranean Sea)**

Alba Medrano^1*^, Bernat Hereu^1^, Simone Mariani^1,2^, João Neiva^3^, Marta Pagès-Escolà^1^, Cristina Paulino^3^, Graciel·la Rovira^1^, Ester A. Serrão^3^, Cristina Linares^1^

^1^ Department of Evolutionary Biology, Ecology and Environmental Sciences, Institut de Recerca de la Biodiversitat (IRBIO), University of Barcelona, Av. Diagonal 643, 08028 Barcelona, Spain

^2^ Centre d’Estudis Avançats de Blanes – CSIC, Accés Cala Sant Francesc 14, 17300 Blanes, Girona, Spain

^3^ Center of Marine Science (CCMAR), University of Algarve, Campus de Gambelas, 8005-139 Faro, Portugal

*Corresponding author: [amedrano@ub.edu](mailto:amedrano@ub.edu)

**Supplementary Table S1.** AMOVA analyses results for differentiation between the three populations. P-value significance for 10,100 permutations.

| **Source of Variance** | **df** | **Sum of Squares** | **Variance Components** | **% Variation** | **F-_ST_** | **P-value** |
| --- | --- | --- | --- | --- | --- | --- |
| Among populations | 2 | 6.41 | 0.074 | 6.7 | 0.07 | **<0.001** |
| Within populations | 85 | 87.84 | 1.033 | 93.3 |  |  |

**Supplementary Table S2**. Proportion of membership of each population (q-values) considering a range of genetic clusters (K) from 1 to 5.

|  | Inferred Clusters | |  |  |  |
| --- | --- | --- | --- | --- | --- |
| Given Pop | 1 | 2 | 3 | 4 | 5 |
| 1 (Begur) | 0.154 | 0.169 | 0.233 | 0.256 | 0.198 |
| 2 (Medes I.) | 0.215 | 0.218 | 0.185 | 0.176 | 0.206 |
| 3 (Messina) | 0.238 | 0.204 | 0.197 | 0.167 | 0.194 |

**Supplementary Table S3.** Pearson correlation coefficients between the length of the longest axis of *T. elegans* and other morphometric variables

| Variable | r2 | p |
| --- | --- | --- |
| Biomass of branchlets (g fw) | 0.61 | **<0.01** |
| Holdfast diameter (cm) | 0.40 | **0.04** |
| Main axis length (cm) | 0.48 | **0.01** |
| Number of tophules | 0.48 | **0.01** |
| Number of primary axes | 0.20 | 0.33 |
